# Estimating vaccine effectiveness in preventing laboratory‐confirmed influenza in outpatient settings in South Africa, 2015

**DOI:** 10.1111/irv.12436

**Published:** 2016-12-20

**Authors:** Johanna M. McAnerney, Sibongile Walaza, Stefano Tempia, Lucille Blumberg, Florette K. Treurnicht, Shabir A. Madhi, Ziyaad Valley‐Omar, Cheryl Cohen

**Affiliations:** ^1^ National Health Laboratory Services (NHLS)National Institute for Communicable Diseases (NICD)JohannesburgSouth Africa; ^2^ Influenza DivisionU.S. Centers for Disease Control and PreventionAtlantaGAUSA; ^3^ Influenza ProgramU.S. Centers for Disease Control and PreventionPretoriaSouth Africa; ^4^ Faculty of Health SciencesMedical Research Council: Respiratory and Meningeal Pathogens Research UnitUniversity of the WitwatersrandJohannesburgSouth Africa; ^5^ Division of Medical VirologyClinical Laboratory SciencesUniversity of Cape TownCape TownSouth Africa

**Keywords:** effectiveness, influenza, vaccine

## Abstract

Trivalent seasonal influenza vaccine effectiveness during the 2015 season in South Africa was assessed using a test‐negative case control study design. Influenza A(H1N1)pdm09 was the dominant circulating strain. Overall influenza vaccine coverage was 3.2% (29/899). The vaccine effectiveness estimate, against any influenza virus infection, adjusted for age, underlying conditions and timing within season was 46.2% (95% CI: −23.5 to 76.5), and 53.6% (95% CI: −62.6 to 80.3) against influenza A(H1N1)pdm09.

## 1 Introduction

South Africa has a long‐standing influenza sentinel surveillance system the Viral Watch which was started in 1984, to describe influenza seasonality and provide influenza strains for global vaccine strain selection. Sites are mainly general practitioners in the private healthcare setting, who submit the majority of specimens during the influenza season. Since 2005, it has also been used to estimate influenza vaccine effectiveness (VE).^1^, ^2^, ^3^, ^4^ Recommendations for the use of influenza vaccine are published annually in South Africa.^5^ Annual vaccination is recommended for individuals at increased risk of complications or healthy individuals wishing to reduce their risk of contracting influenza. Since 2010, the South African Department of Health has conducted annual influenza vaccination campaigns. For the South African population of ≈55 million, 820 000 doses were used in the public sector serving 80% of the population with an estimated 20 million persons in the at risk group.^6^ In addition, in the private sector approximately 1 million doses are used annually for the remaining 20% of the South African population covered by health insurance. The influenza vaccine strains included in the 2015 vaccine in South Africa were as follows: A/California/7/2009 (H1N1)‐like virus, A/Switzerland/9715293/2013 (H3N2)‐like virus and B/Phuket/3073/2013‐like virus (Yamagata lineage). We aimed to estimate trivalent influenza vaccine (TIV) effectiveness against laboratory‐confirmed medically attended influenza illness for the 2015 influenza season in South Africa and characterise circulating strains.

## 2 Methods

During 2015, 107 outpatient practitioners at 67 practices in eight of the nine provinces of South Africa participated in the Viral Watch sentinel influenza surveillance programme. Patients presenting with influenza‐like illness (ILI) to participating practitioners and testing influenza virus‐positive were defined as cases, whereas those who tested negative were used as controls. ILI was defined as acute respiratory illness with a measured temperature of ≥38°C or a history of fever, and cough, with onset within the past 10 days. Throat and/or nasal swabs were taken from a maximum of five patients per week, at the practitioner's discretion, as part of routine diagnostic investigations for which informed written consent was not required.

Specimens were tested using multiplex reverse transcription real‐time polymerase chain reaction (rRT‐PCR) assays for influenza A and B. Influenza A‐positive specimens were further subtyped by rRT‐PCR.^7^ Clinical, demographic and influenza vaccination data were collected from each patient at the time of specimen collection. Patients aged ≥6 months meeting the ILI case definition with available influenza vaccine history were included in the VE analysis. Vaccine history was self‐reported or from provider records, where available, and it was not recorded whether children <9 years had received two doses. Patients who had received the current season influenza vaccine ≥14 days prior to the onset of illness were considered vaccinated. Patients who had received influenza vaccine <14 days prior to onset of symptoms were excluded. Underlying conditions collected were as follows: chronic pulmonary and cardiac disease, immunosuppression (including HIV), metabolic disorders, pregnancy and morbid obesity defined as a body mass index of ≥40.

The start of the influenza season was defined as two consecutive weekly influenza detection rates of ≥10%, and the end as when the detection rate dropped below 10% for two consecutive weeks, or <10 specimens per week were received.^1^ The season was divided into three equal parts as follows: early (weeks 19‐24); mid (weeks 25‐31); late (weeks 32‐37). Only specimens collected during the season were included in the VE analysis. Multivariate logistic regression was used to adjust VE estimates by age, pre‐existing underlying medical conditions and timing within season. Vaccine effectiveness was calculated as 1‐odds ratio (OR) for laboratory‐confirmed influenza in vaccinated and unvaccinated patients. All analyses were conducted using Stata version 14 (StataCorp LP, College Station, TX, USA).

## 3 Results

**Figure 1:**
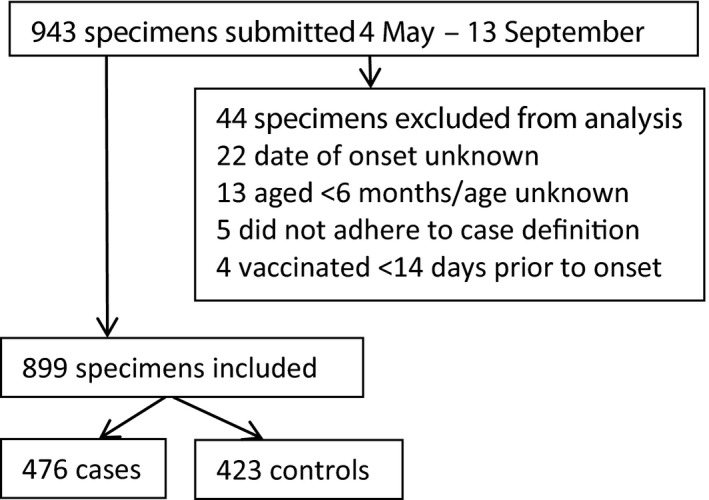
Flow of specimens and allocation of cases and controls for VE analysis, South Africa 2015

**Figure 2:**
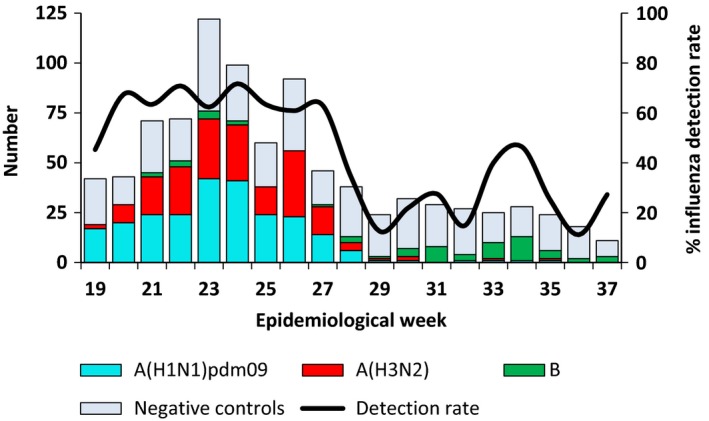
Test‐negative controls and laboratory‐confirmed cases by week and virus subtype: Viral Watch programme, South Africa, 4 May‐13 September 2015 [Colour figure can be viewed at wileyonlinelibrary.com]

The 2015 influenza season in South Africa started in week 16 (week ending 19 April) and ended in week 37 (week ending 13 September). As the vaccine only became available in week 17, we restricted our analysis to weeks 19 to 37. During this time, 943 individuals were enrolled and tested and of whom 899 (95.3%) were eligible for the VE analysis. Amongst the patients excluded there were four who had received influenza vaccine <14 days prior to onset of symptoms. (Figure 1) The overall influenza detection rate was 52.9% (476/899) amongst individuals included. The majority of influenza detections were influenza A(H1N1)pdm09 which accounted for 242/476 (50.8%) of the total influenza subtypes detected, followed by influenza A(H3N2) which accounted for 182/476 (38.2%) of detections with the remaining detections being influenza B which occurred in low numbers throughout the season. (Figure 2) All influenza A(H1N1)pdm09 viruses detected were in the 6B genetic lineage and continued drift was observed, whereas almost all influenza A(H3N2) viruses were in the 3C.2a genetic lineage. Influenza B viruses identified in 2015 were in clade 3 of the B/Yamagata lineage.

The majority [599/899 (66.6%)] were patients aged 18‐64 years, and 477 (53.1%) patients were female. Fifty per cent (448/899) of specimens were collected in the early weeks of the season, although this proportion was higher [288/476 (60.5%)] for cases. The majority of specimens [805/899 (89.5%)] were collected within 3 days of symptom onset. Pre‐existing underlying medical conditions were reported in 127/899 (14.1%) patients. (Table 1).

**Table 1:** Characteristics of cases (influenza test‐positive) and controls (influenza test‐negative) in the Viral Watch programme, South Africa, 2015

Variable	Cases N=476	Controls N=423	Total N=899	*P*
	n (%)	n (%)	n (%)	
Vaccine receipt				
Vaccinated	9 (1.9)	20 (4.7)	29 (3.2)	.02
Not vaccinated	467 (98.1)	403 (95.3)	870 (96.8)	
Age group				
Median	32 y	33 y	32 y	.04
<18 y	143 (30.0)	112 (26.5)	255 (28.4)	
18‐64 y	317 (66.6)	282 (66.7)	599 (66.6)	
≥65 y	16 (3.4)	29 (6.9)	45 (5.0)	
Sex				
Male	230 (48.3)	186 (44.0)	416 (46.3)	.32
Female	242 (50.8)	235 (55.6)	477 (53.1)	
Unknown	4 (0.8)	2 (0.5)	6 (0.6)	
Seasonality				
Early (weeks 19‐24)	288 (60.5)	160 (37.8)	448 (49.9)	<.01
Mid (weeks 25‐31)	152 (31.9)	167 (39.5)	319 (35.5)	
Late (weeks 32‐37)	36 (7.6)	96 (22.7)	132 (14.7)	
Region				
Central Plateau^a^	211 (44.3)	221 (52.3)	432 (48.1)	<.01
North East Subtropical^b^	70 (14.7)	72 (17.0)	142 (15.8)	
Southern coastal belt^c^	195 (41.0)	130 (30.7)	325 (36.1)	
Underlying condition^d^				
None	404 (84.9)	368 (87.0)	772 (85.9)	.37
Yes	72 (15.1)	55 (13.0)	127 (14.1)	
Interval between onset and sampling (days)				
0‐3 d	440 (92.4)	365 (86.3)	805 (89.5)	.03
4‐10 d	36 (7.6)	58 (13.7)	94 (10.5)	

a Free State, Gauteng, Northern Cape and North West Provinces.

b Mpumalanga and Limpopo Provinces.

c Eastern Cape and Western Cape Provinces.

d Provinces grouped into 3 regions

Overall, the influenza vaccine coverage was 1.9% in cases (9/476) and 4.7% (20/423) in controls (*P*=.02). Coverage in patients with underlying conditions was 4.2% (3/72) in cases and 5.5% (3/55) in controls (*P*=.79) and in those aged ≥65 years was 12.5% (2/16) in cases and 3.2% (1/31) in controls (*P*=.39), but numbers were small. (Table 2) Of the nine vaccinated influenza‐positive patients, five were positive for influenza A(H1N1)pdm09, and two each for influenza A(H3N2) and influenza B.

**Table 2:** Vaccine receipt and vaccine effectiveness (VE) estimates by presence of underlying medical conditions (UMC) and age group and timing within season, Viral Watch programme, South Africa, 2015

	Vaccine coverage			Percentage Unadjusted VE (95% CI)
	Cases	Controls	Total	
	n/N (%)	n/N (%)	n/N (%)	
Total	9/476 (1.9)	20/423 (4.7)	29/899 (3.2)	61.2 (13.8, 82.5)
UMC^a^	3/72 (4.2)	3/55 (5.5)	6/127 (4.7)	24.6 (−288.6, 85.4)
No UMC	6/405 (1.5)	17/368 (4.6)	23/772 (3.0)	68.9 (20.2, 87.9)
<18 y	0/143 (0)	1/112 (0.9)	1/255 (0.4)	
18‐64 y	7/317 (2.2)	18/282 (6.4)	25/599 (4.2)	66.9 (19.5, 86.4)
≥65 y	2/16 (12.5)	1/29 (3.4)	3/45 (6.7)	−300.0 (−4699.1, 66.7)
Central Plateau	4/211 (1.9)	14/221 (6.3)	18/432 (4.2)	71.4 (11.7, 90.7)
NE Subtropical	4/70 (5.7)	1/72 (1.4)	8/142 (5.6)	−330.3 (−3849.2, 53.1)
Southern coastal	1/195 (0.5)	5/130 (3.8)	6/325 (1.8)	87.1 (−11.6, 85.1)
Season: early	1/288 (0.3)	3/160 (1.9)	4/448 (0.9)	81.7 (−76.8, 98.1)
Season: mid	7/152 (4.6)	11/167 (6.6)	18/319 (5.6)	31.5 (−81.4, 74.2)
Season: late	1/36 (2.8)	6/96 (6.3)	7/132 (5.3)	57.1 (−268.9, 95.0)

a Underlying medical conditions: chronic pulmonary and cardiac disease, immunosuppression (including HIV), metabolic disorders, pregnancy, and morbid obesity defined as a body mass index of ≥40.

Vaccine effectiveness estimates for all influenza adjusted for possible confounding factors showed timing within season to be the major confounder. (Table 3).

**Table 3:** Vaccine effectiveness (VE) estimates (all influenza) adjusted for possible confounding factors, Viral Watch programme, South Africa, 2015

Adjustment variable	Percentage adjusted VE (95% CI)
Underlying medical conditions	61.7 (14.9, 82.8)
Age (<18 y; 19‐64 y; ≥65 y)	58.9 (8.1, 81.6)
Age (6‐59 mo, 5‐19 y, 20‐44 y, 45‐64 y, ≥65 y)	59.5 (9.3, 81.9)
Season (early, mid, late)	48.3 (−17.6, 77.3)
Collection after onset (≤3 d; 4‐10 d)	65.2 (14.3, 82.7)
Region (Central plateau; North East subtropical; Southern coastal belt)	58.7 (7.9, 81.5)

The overall VE estimate, adjusted for age, underlying conditions, and timing within season, was 46.2% (95% CI: −23.5 to 76.5) against any influenza virus type, 53.5% (95% CI: −62.6 to 80.3) against influenza A(H1N1)pdm09, 65.9% (95% CI: −53.9 to 92.4) against influenza A(H3N2) and 33.0% (95% CI: −207.8 to 85.4) against any lineage of influenza B. When restricted to specimens collected within 3 days of symptom onset, VE against any influenza, and influenza A(H3N2) increased, but decreased for influenza A(H1N1)pdm09 and influenza B. When restricted to the weeks that the type or subtype was circulating VE only decreased for influenza A(H1N1)pdm09.(Table 4).

**Table 4:** Vaccine receipt and vaccine effectiveness by influenza type and subtype adjusted by age, underlying conditions and season

Influenza type/subtype	Vaccine coverage			Percentage adjusted VE
	Cases	Controls	Total	
	n/N (%)	n/N (%)	n/N (%)	
All specimens				
Any influenza	9/476 (1.9)	20/423 (4.7)	29/899 (3.2)	46.2 (−23.5, 76.5)
A(H1N1)pdm09	5/242 (2.1)	20/423 (4.7)	25/665 (3.8)	53.5 (−62.6, 80.3)
A(H3N2)	2/182 (1.1)	20/423 (4.7)	22/605 (3.6)	65.9 (−53.9, 92.4)
B	2/57 (3.5)	20/423 (4.7)	22/480 (4.6)	33.0 (−207.8, 85.4)
Specimens collected ≤3 d after onset of symptoms				
Any influenza	8/440 (1.8)	18/365 (4.9)	26/805 (3.2)	52.2 (−15.0, 80.1)
A(H1N1)pdm09	5/225 (2.2)	18/365 (4.9)	23/590 (3.9)	43.9 (−63.1, 80.7)
A(H3N2)	1/165 (0.6)	18/365 (4.9)	19/530 (3.6)	82.1 (−39.8, 77.1)
B	2/55 (3.6)	18/365 (4.9)	20/420 (4.8)	32.0 (−216.4, 85.4)
Only weeks when type/subtype was circulating				
A(H1N1)pdm09	5/242 (2.1)	16/378 (4.2)	21/620 (3.4)	37.2 (−85.9, 78.8)
A(H3N2)	2/182 (1.1)	13/341 (3.8)	15/523 (2.9)	61.3 (−79.4, 91.6)
B	2/57 (3.5)	20/423 (4.7)	22/480 (4.6)	36.5 (−194.1, 86.3)

Vaccine effectiveness adjusted for underlying conditions and timing within seasons for adults aged 18 to 64 years for any influenza was 54.4% (95% CI: −14.1 to 81.8), 37.3% (95% CI: −93.6 to 77.7) against influenza A(H1N1)pdm09 and 28.2% (95% CI: −236.5 to 84.7) against influenza B. When restricted to specimens collected within 3 days of onset, or when the type or subtype was circulating, a decrease in VE was shown in both occasions for influenza A(H1N1)pdm09. None of the cases positive for influenza A(H3N2) in this age group had received vaccine. (Table 5).

**Table 5:** Vaccine receipt and vaccine effectiveness in adults aged 18‐64 y adjusted by underlying conditions and season

Influenza type/subtype	Vaccine coverage			Percentage adjusted VE
	Cases	Controls	Total	
	n/N (%)	n/N (%)	n/N (%)	
All specimens				
Any influenza	7/317 (2.2)	18/282 (6.4)	25/599 (4.2)	54.4 (−14.1, 81.8)
A(H1N1)pdm09	5/181 (2.8)	18/282 (6.4)	23/463 (5.0)	37.3 (−93.6, 77.7)
B	2/34 (5.9)	18/282 (6.4)	20/316 (6.3)	28.2 (−236.5, 84.7)
Specimens collected ≤3 d after onset of symptoms				
Any influenza	7/302 (2.3)	16/263 (6.1)	23/565 (4.1)	53.5 (−18.4, 81.8)
A(H1N1)pdm09	5/182 (2.7)	16/263 (6.1)	21/445 (4.7)	34.5 (−96.1, 78.1)
B	2/37 (5.4)	16/263 (6.1)	18/300 (6.0)	25.4 (−255.3, 84.3)
Only weeks when type/subtype was circulating				
A(H1N1)pdm09	5/181 (2.8)	14/239 (5.9)	19/420 (4.5)	25.7 (−127.0, 75.7)
B	2/37 (5.4)	18/282 (6.4)	20/319 (6.3)	35.7 (−200.2, 86.2)

## 4 Discussion

Influenza A(H1N1)pdm09 which accounted for the majority of influenza detections, circulated simultaneously with influenza A(H3N2) during the season. Sporadic detections of influenza B were made from week 21 (week ending 24 May), but the majority of influenza B detections were made at the end of the season continuing until the end of December. Our VE results suggest that overall influenza vaccine was 46% effective in preventing laboratory‐confirmed influenza in our setting. Point estimates per type/subtype adjusted for age and underlying medical conditions ranged from 33% against influenza B to 66% against influenza A(H3N2) with wide confidence intervals.

The influenza season started early in week 16 (week starting 13 April). Due to technical difficulties, the vaccine was only available from late April onwards which may have compounded the low influenza vaccine coverage of 3%, although overall vaccine coverage in previous years in the same population has ranged from 1.8% in 2012 to 5.1% in 2006.^2^, ^3^, ^4^ In the early part of the season, four patients had to be excluded from the analysis due to having received influenza vaccine <14 days prior to onset of symptoms.

Amongst other southern hemisphere countries, New Zealand reported preliminary overall VE of 36% against ILI, and a VE of 50% against hospitalisation in a season where A(H3N2) predominated.^8^ In Australia, influenza B predominated changing from B/Yamagata lineage in the early season to B/Victoria lineage in the latter part of the season.^9^ Early VE against medically attended laboratory‐confirmed influenza for the first 10 weeks of the season in Europe was reported to be 46% with a predominance of influenza A(H1N1)pdm09.^10^

Although persons aged ≥65 years had the highest vaccine coverage, we were unable to show VE in this age group due to the small sample size. Previous studies have shown that although antibody response and protection elicited by influenza vaccination are lower amongst the elderly, influenza vaccination in this group is still associated with reductions in the rates of hospitalisation and death.^11^, ^12^ In addition, the percentage increase in winter deaths attributable to influenza was substantially higher in South African elderly as compared to the United States.^13^

There are several limitations to our study especially the low vaccine coverage which affected the ability to statistically estimate significance of VE amongst subgroups such as individuals >65 years of age. Although the VE point estimates varied when analysed restricted by time of specimen collection after onset, or weeks when the type or subtype was circulating and none were statistically significant, we cannot exclude the potential of residual confounding. In addition, Viral Watch patients are unlikely to be a random sample, and the vast majority are patients accessing private health care, whereas only about 20% of the South African population have private healthcare insurance; however, they are also the group with highest influenza vaccine coverage. Influenza vaccination status and underlying conditions were self‐reported by some patients to the practitioner, which could have led to misclassification.

## 5 Conclusion

Despite low influenza vaccine coverage in South Africa, we were able to estimate VE. Late arrival of the vaccine may have contributed to limiting the number of patients protected against influenza during the season. Influenza vaccine had moderate effectiveness in our setting in 2015.
